# Stakeholder perspectives of mental healthcare services in Bangladesh, its challenges and opportunities: a qualitative study

**DOI:** 10.1017/gmh.2024.30

**Published:** 2024-03-12

**Authors:** Kamrun Nahar Koly, Jobaida Saba, Mala Rao, Sabrina Rasheed, Daniel D. Reidpath, Stephanie Armstrong, Shamini Gnani

**Affiliations:** 1 Health System & Population Studies Division, International Centre for Diarrhoeal Disease Research, Bangladesh (icddr,b), Dhaka, Bangladesh; 2 Department of Primary Care and Public Health, School of Public Health, Faculty of Medicine, Imperial College London, London, UK; 3 Jeffrey Cheah School of Medicine and Health Sciences, Monash University Malaysia, Subang Jaya, Malaysia; 4 School of Health and Social Care, College of Health and Science, University of Lincoln, Lincoln, UK

**Keywords:** mental health, mental health system, mental healthcare pathways, mental healthcare-seeking behaviour, Bangladesh

## Abstract

This study explores Bangladesh’s mental health services from an individual- and system-level perspective and provides insights and recommendations for strengthening it’s mental health system. We conducted 13 in-depth interviews and 2 focus group discussions. Thirty-one participants were recruited using a combination of purposive and snowball sampling methods. All interviews and group discussions were audio-recorded and transcribed, and key findings were translated from Bengali to English. Data were coded manually and analysed using a thematic and narrative analysis approach. Stakeholders perceived scarcity of service availability at the peripheral level, shortage of professionals, weak referral systems, lack of policy implementation and regulatory mechanisms were significant challenges to the mental health system in Bangladesh. At the population level, low levels of mental health literacy, high societal stigma, and treatment costs were barriers to accessing mental healthcare. Key recommendations included increasing the number of mental health workers and capacity building, strengthening regulatory mechanisms to enhance the quality of care within the health systems, and raising awareness about mental health. Introducing measures that relate to tackling stigma, mental health literacy as well as building the capacity of the health workforce and governance systems will help ensure universal mental health coverage.

## Impact statement

Bangladesh is a low- to middle-income country that has a high burden of poor mental health. Low education levels, poverty, hunger, chronic underlying medical issues, family history and natural catastrophes are associated factors for poor mental health in Bangladesh. The mental healthcare system is unable to provide care all over the country. This is the first study that explores the individual and system-level challenges in providing universal mental healthcare in Bangladesh and combines recommendations articulated from stakeholders’ perspectives. This qualitative study involved 31 participants, including psychiatrists, psychologists, mental health advocates and people with lived experiences of mental health conditions. This study has the potential to guide policymakers and relevant authorities to focus on programmes at the individual level (low mental health literacy and stigma) and also at the system level (low availability of services and professionals in remote areas, lack of investments, and absence of monitoring and regulatory bodies to ensure quality of care).

## Introduction

Mental health disorders represent a significant proportion of the global burden of disease, affecting people of all ages, cultures and socioeconomic levels (Borges et al., 2010). More than 70% of the worldwide burden of mental disorders lies in low- and middle-income countries (LMICs) like Bangladesh, yet investment in mental healthcare services is low (Lopez et al., 2006; Jacob et al., 2007; Tomlinson, 2013). Poor mental health in Bangladesh is associated with a wide range of factors, such as low levels of education, poverty, malnutrition, long-term medical conditions, family history and natural disasters (Hossain et al., 2014; Hasan et al., 2022; Tasdik Hasan et al., 2020; Alam et al., 2021).

A systematic review of prevalence estimates of mental health conditions in Bangladesh between 1975 and 2013 ranged from 6.5% to 31% among adults and 13.4% to 22.9% among children (Hossain et al., 2014; Ghebreyesus, 2019). The National Mental Health Survey of Bangladesh in 2019 estimated that 16.8% of adults and 13.6% of children had a mental health condition, and of concern was that more than 90% of adults and children did not seek treatment (WHO, 2019). Considering the population’s prevailing levels of mental health conditions, the mental health treatment gap remains egregious.

Despite this extreme treatment gap, mental healthcare in Bangladesh is not a clear component of its Universal Health Coverage or covered through a national social health insurance system. Consequently, individuals pay for mental healthcare themselves; and about 64% of mental healthcare expenditure is out-of-pocket (World Health Organization. Regional Office for the Western Pacific, 2015). Furthermore, most mental healthcare facilities are clustered in urban areas, especially in metro cities, despite 70% of the 163 million population living in rural areas. There is one national specialist level mental health treatment and research facility centre (National Institute of Mental Health, NIMH) in the capital Dhaka; a specialised 500 bedded mental health hospital in Pabna district in the northwest region, and a total of 69 mental health hospitals outpatient facilities across the country (Choudhury et al., 2006; Islam and Biswas, 2015). There are no mental health services at a primary care or community level. According to recent World Health Organization (WHO) reports for 2020, Bangladesh has an estimated 1.17 mental health professionals available per 100,000 population, 270 psychiatrists, 565 psychologists and other paid mental health workers that work in government and non-government organisations (NGOs). There are 700 general nurses (0.4 per 100,000) who work in the two specialised government mental health hospitals: NIMH and Pabna Mental Hospital. More than 50% of psychiatrists work in both the public and private sectors, and there are only six child psychiatrists to provide services for the entire population (WHO, 2022).

In Bangladesh, limited research has been undertaken that examines mental healthcare services from the perspective of stakeholders, for example, those with a lived experience of poor mental health and from outside the setting of a specialist mental health hospital (Nuri et al., 2018). Our study was designed to explore how mental health services in Bangladesh are being used, the challenges faced by those using and providing services, and the perceived solutions on how the mental health system could be strengthened at an individual and systems level.

## Methods

### Study design

We used qualitative research methodologies to investigate the mental health system in Bangladesh. Our aim was to gain a deeper understanding of how people access care for mental health conditions from the perspective of those who use and provide the services and, as such, is well suited to a qualitative methodological approach (Stebbins, 2001). We conducted in-depth interviews (IDIs) and focus group discussions (FGDs) to explore stakeholders’ views and adopted the inductive narrative analysis approach (McAllum et al., 2019) to formulate recommendations for strengthening the mental health system. IDIs provide a more private space to explore and uncover personal opinions without individuals experiencing fear of judgment by others (Setia, 2017). FGDs were used to understand the perspective of access to mental health care by participants who shared corresponding and contrasting opinions (Bloor, 2001). The knowledge gleaned from interviews and FGDs was used to support the generation of recommendations (Kumar, 1989). The study data were analysed using the thematic analysis approach, which offers a comprehensive, intricate, and multifaceted description of the data via searching and identifying common themes (Braun and Clarke, 2012; Vaismoradi et al., 2013).

### Study participants and sampling

We used purposive and snowball sampling to recruit participants for interviews and FGDs. Participants were either involved in providing mental health services (psychiatrists, psychologists and mental health advocates from civil society and non-governmental organisations) or had a lived experience of poor mental health and used health services; this ensured that we recruited participants with diverse experiences accessing care and providing mental health services (Marshall, 1996; Shaheen and Pradhan, 2019). Psychiatrists and psychologists were recruited pragmatically from both private and public sectors. We identified mental health advocates using a combination of strategies that included their social media activities, their mental health advocacy role, and their role in providing community-level mental health services. We used service providers and civil society organisations to help identify people with a lived experience of mental health conditions.

Psychiatrists are recognised as providers of governmental mental healthcare, unlike psychologists and provision of mental health services also takes place in the non-governmental sector. We conducted two focus groups, one among nine psychologists and one among nine mental health advocates, to gather a range of opinions on the experience of mental healthcare provision in Bangladesh and possible solutions to strengthen the mental health system. Data collection took place during the coronavirus disease-2019 (COVID-19) pandemic when social distancing measures and restrictions were in place, and therefore, the FGDs were conducted online and in preference as participants were working from home. Our intention had been to undertake an FGD with psychiatrists but due to issues of feasibility resulting from their shift work in the public sector and private work, which was challenging during the pandemic, we were unable to do so and instead conducted individual in-depth interviews with six psychiatrists. Additionally, we conducted in-depth interviews in preference to FGDs with seven persons with lived experience of mental health to reduce any psychological harm and maintain privacy and confidentiality over their mental health journey. The overall number of interviews and FGDs we undertook was influenced by the COVID-19 pandemic; however, we were still able to collect rich sources of data for analysis.

### Data collection

Four semi-structured topic guides were developed for each type of stakeholder group in conducting in-depth interviews and FGDs (Supplementary 1 of the Supplementary Material). The topic guides covered areas of mental health service provision, care-seeking patterns, challenges in providing services, and recommendations for strengthening the mental health system. The interview topic guides were developed based on prior studies conducted in similar country settings such as Nepal, Uganda and China (Nsereko et al., 2011; Brenman et al., 2014; Ren et al., 2018; Tol et al., 2018). Moreover, it was modified after piloting with three participants (a psychiatrist, a psychologist and a person with lived experience of mental health condition), and new questions were added to explore further areas and ensure better probing and analysis. Data collection took place more than 4 months, from September to December 2020.

Participants were invited to participate in the study by telephone, followed by an e-mail invitation sent by the lead author with sharing a personal introduction, the purpose of the study, and those who consented were enrolled. None of the participants refused to participate in the study. All interviews and FGDs were conducted and recorded online using Microsoft Teams software. The participants were asked to be in a private space during the interview to ensure confidentiality. Each interview lasted 40–50 min, and FGDs lasted 110–120 min. The lead author conducted all interviews and moderated the FGDs; for the FGDs, two additional researchers took field notes. There were no repeated interviews. All three researchers had previous experience collecting qualitative data and received supplementary training in FGD methodology and qualitative data collection and analysis. All interviews and FGDs were conducted and transcribed in Bangla, with important findings translated into English. The transcripts were checked by the co-investigators for quality and consistency.

### Data analysis

Study data were analysed using inductive thematic analysis (Braun and Clarke, 2012). Transcripts were manually and individually coded following iterative reading in Microsoft Excel. Data were separated under each code heading, and common themes and sub-themes were examined in relation to the study aims. Each theme was reviewed, and a thematic matrix was constructed in Microsoft Excel for further analysis. Three researchers cross-checked coded transcripts and any discrepancies in coding were resolved.

Themes and sub-themes were developed and coded with a separate data sheet of quotes assigned to each relevant theme and sub-theme. These were compared and triangulated, and regular peer discussions on comprehension and data interpretation by the study team were held in more than ten (1-hour) meeting sessions. We adhered to the consolidated reporting criteria for qualitative studies (COREQ; Supplementary 2 of the Supplementary Material; Tong et al., 2007).

### Ethical statement

Ethics approval was sought by the Institutional Review Board (IRB) of icddr, b, Bangladesh (PR 20094). Informed consent was obtained from all participants, and the study was carried out in accordance with the Institutional Research Ethics guidelines and ethical guidelines involving human participation (Goodyear et al., 2007).

## Results

A total of 31 stakeholders participated in our study (Table 1). All the psychiatrists were male, with at least 10 years of clinical experience, and four having more than 15 years of experience providing mental health services in the public and private sectors (Table 2). Five psychiatrists were based in a public organisation and held senior leadership positions. Among the psychologists, seven were female and were academically trained in “clinical psychology” and “educational and counselling psychology”; five of the psychologists had 10–15 years of experience in counselling and five worked for private organisations. Among the mental health advocates, seven were female, had 10–15 years of experience in mental health advocacy, and held affiliations in mental health service provision. The majority of the people with lived experience of mental health illnesses were women, aged from 24 to 53 years and had experienced a treatment gap of between 1 month and 13 years (Table 3).

**Table 1.** Qualitative method and sample size

Methods	Type of stakeholders	Gender		Total number
		Male	Female	
IDI (in-depth interviews)	Psychiatrists	6	0	6
	People with lived experiences	2	5	7
FGD (focus group discussion)	Psychologists	1	8	9
	Mental health advocates	2	7	9
				31

**Table 2.** Information about mental health service providers

Type of stakeholder	Participants ID	Gender	Occupation and place of work	Type of organisation	Years of experience in the mental health sector
Psychiatrists	Psychiatrist 1	Male	Psychiatrist	Both government and non–government tertiary level facility	10 years
	Psychiatrist 2	Male	Associate professor	Both government and non–government tertiary level facility	15 years
	Psychiatrist 3	Male	Assistant professor	Both government and non–government tertiary level facility	10 years
	Psychiatrist 4	Male	Psychiatrist, professor	Both Government and non–government tertiary level facility	15 years
	Psychiatrist 5	Male	Psychiatrist, professor	Both government and non–government tertiary level facility	20 years
	Psychiatrist 6	Male	Psychiatrist	Private facility	24 years
Psychologist	Psychologist 1	Female	Counselling psychologist, professor	Governmental Educational Institution	25 years
	Psychologist 2	Male	Clinical psychologist, professor	Governmental Educational Institution	22 years
	Psychologist 3	Female	Clinical psychologist, associate professor	Governmental educational institution	15 years
	Psychologist 4	Female	Educational psychologist, associate professor	Governmental educational institution	10 years
	Psychologist 5	Female	Psychosocial counsellor and lecturer	Non–governmental educational institution	10 years
	Psychologist 6	Female	Counselling psychologist, consultant	Non– governmental institution	8 years
	Psychologist 7	Female	Psychotherapist, director	Non– governmental institution	9 years
	Psychologist 8	Female	Counselling psychologist, psychosocial counsellor and lecturer	Non– governmental educational institution	9 years
	Psychologist 9	Female	Counselling psychologist, consultant	Non– governmental institution	8 years
Mental health advocates	Mental health advocate 1	Female	Counsellor, health service-providing organisation	Non–government al institution	10 years
	Mental health advocate 2	Female	Founder, lead consultant of a mental health service-providing organisation	Non–government al institution	10 years
	Mental health advocate 3	Female	Educational psychologist, of a mental health service-providing organisation	Non–government al institution	12 years
	Mental health advocate 4	Male	Counsellor of a mental health service-providing organisation	Non–governmental institution	7 years
	Mental health advocate 5	Female	Mental health counsellor of an online mental health service-providing organisation	Non–governmental institution	10 years
	Mental health advocate 6	Female	Founder of a mental health service-providing organisation	Non–governmental institution	10 years
	Mental health advocate 7	Male	Mental health researcher	Non–governmental institution	5 years
	Mental health advocate 8	Female	Mental health practitioner	Non–governmental institution	5 years
	Mental health advocate 9	Female	Mental health researcher	Non–governmental institution	18 years

**Table 3.** Information on persons with lived experiences of mental health conditions

Type of stakeholder	Participant Id	Age	Gender	Mental health conditions	Experienced treatment gap
Persons with lived experiences of mental health conditions	PWLE 1	26	Female	Anxiety, suicidal ideation	13 years later
	PWLE 2	24	Male	Low energy, persistent sadness	5/6 years
	PWLE 3	24	Female	Emotionally overwhelmed, anger, sadness, lack of hope, suicidal ideation	5/6 years
	PWLE 4	27	Female	Breathing problems, stress, trauma, sleep difficulties, emotional breakdown	2 months
	PWLE 5	29	Female	Emotional breakdown, breathing problems, problems in concentration, eating disorder for several days, panic attack	1 year
	PWLE 6	48	Male	Hyperactive, substance abuse, addiction	6 years
	PWLE 7	53	Female	Restlessness, panic attack, nothing feels good, beating children, anger, breaking things	6 years and then 13 years

**Figure 1.**
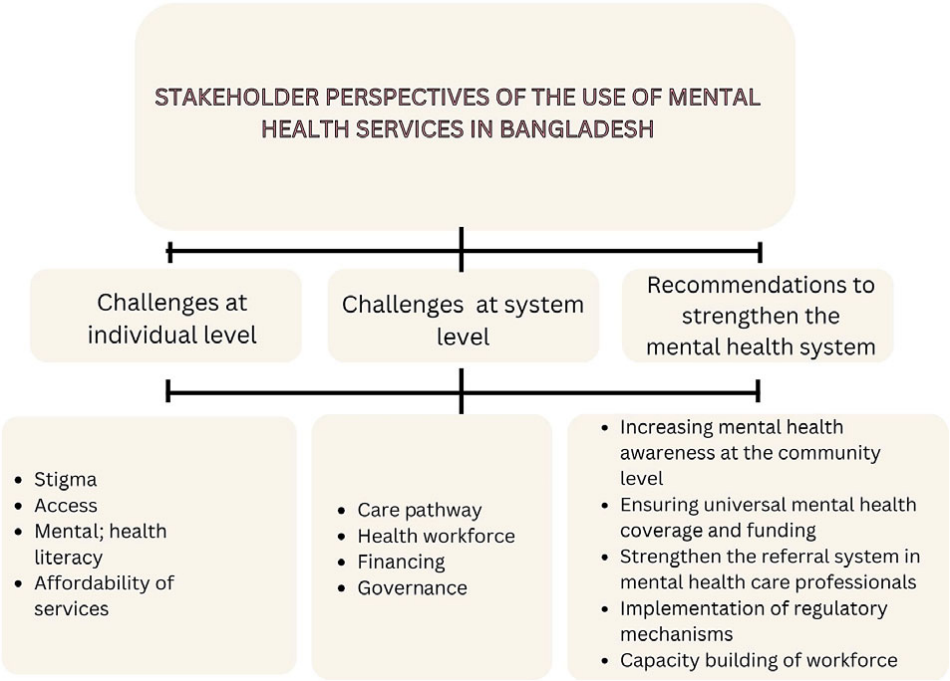
Thematic analysis of the findings.

We present combined data from interviews and FGDs, which yielded rich data that were grouped across three areas: from the perspective of the individual with a mental health problem seeking treatment, the system challenges in the provision of mental health services, and recommendations on how to strengthen the overall mental health system (Figure 1). To ensure that the reporting and interpretation of the data were a true reflection, all research team members reviewed and agreed on the quotes and contextual text. Further associated quotes are in Supplementary 3 of the Supplementary Material.

## Theme 1: Individual challenges and experience of using mental health services

We identified four sub-themes that relate to how individuals with mental health problems sought care and treatment. These themes covered stigma, mental health literacy, access and affordability to mental health services.

### (I) Stigma

Participants with a lived experience of mental illness spoke powerfully of the impact of stigma, prejudice, and a general lack of awareness within society of mental health problems. This meant that conditions were commonly concealed from their family, work colleagues and social networks. They reported that a physical condition was easier to accept, while a mental health problem was associated with a sense of shame, which meant they received little support in seeking treatment for mental health problems either at home or in the workplace.

As one person with a lived experience (PWLE), who had been in treatment for 5 years, explained:

*I was suggested bed rest for two weeks and diagnosed with panic attacks…I did not understand then…I took leave from work based on a doctor’s prescription. Everyone (colleagues) made fun of me and joked about having a panic attack. PWLE 3*

Participants who were healthcare professionals and mental health advocates also observed similar negative attitudes and behaviours by family members towards individuals who sought care. The consequence meant that treatment was delayed and that this was more pronounced for women. According to one psychologist:

*Clients seek mental health care secretly as most of them fear the judgmental attitude of other family members if they get to know about it. Psychologist 4*

Another female participant stated:

*As a woman, I could not express that I need mental health support from a professional. If I disclose this to my family, they will not be able to marry me off. Society does not want to seek a female with a mental health condition. PWLE 4*

Some participants with poor mental health had even experienced active family pressure not to seek help or stop care. One described this as:

*I started seeking mental health care in 2017 after 14 years of identifying initial symptoms by a general doctor. So far, I have taken only two sessions because my family disapproved. PWLE 1*

A possible explanation for the stigma associated with delays in treatment was the belief that the symptoms of poor mental health were linked to supernatural causes. This meant that people, especially in rural areas, often sought treatment first from traditional or religious healers, which often complicated their mental health status and, in some cases, led to the individuals’ experiencing physical harm. One service provider mentioned:

*Due to a lack of awareness, some local healers victimise rural people by addressing supernatural (Jinn or Pori) possession. Recently, a client who visited me was drenched in blood whom the local healer had beaten. Psychiatrist 3*

### (II) Mental health literacy

Participants reported that stigma was also linked to mental health literacy, the ability to recognise specific disorders or types of psychological distress, the knowledge and beliefs about risk factors and causes, self-help methods, and availability of professional help (Jorm, 2000) and that literacy was low in Bangladesh. The consequence was significant delays in people seeking treatment; participants reported a delay of 6–13 years. As one mental health advocate and provider of services noted:

*People are unaware of the difference between mental health conditions and illnesses. They experienced poor mental health for years but didn’t consider seeking help. People should understand that seeking mental health care does not imply that they are crazy. Mental health advocate 4.*

Intricately linked to people’s level of mental health literacy was knowledge about the nature of treatment for a mental illness. The majority of those with a lived experience of mental illness did not adhere to the treatment, which reflected their lack of trust in the process of accessing care. The participants who were mental health professionals agreed that due to low mental health literacy, people share low trust in the standard mental health treatment. One of the psychiatrists mentioned:

*After taking medication, many people think they are well enough to stop treatment without prior consultation. There is a misconception in society that if antipsychotic medications are taken for a long time, one might become crazy; this misconception largely affects the treatment procedure. Psychiatrist 1*

*Almost all my clients do not complete the sessions if they feel well after the first 1 or 2 sessions. They don’t come further to seek help; then again, when the issue reappears, they come to visit us. Psychologist 1*

Moreover, low literacy dictates their attitude towards psychiatric medication and results, negatively affecting treatment adherence and therapeutic alliance. Furthermore, the treatment delay is also associated with visiting informal care providers such as traditional healers over a proper mental health professional in Bangladesh.

### (III) Access to mental health services

Due to poor levels of mental health literacy and inadequate understanding of mental health illness and treatment, individuals were left to their devices in researching their mental health condition in terms of their physical health, which worsened their condition and had a catastrophic effect on individual productivity and quality of life. Furthermore, they had little understanding of the roles of mental health professionals, and this was a barrier to accessing services. This meant that access to mental health services usually occurred through a referral made by a doctor with whom the individual had consulted for either a physical symptom (e.g., shortness of breath, headache, or sleeping problem) or through networks of relatives, friends and acquaintances who knew or had been treatment by the mental health service provider. This was often due to displays of extreme erratic behaviour. Participants described that people only sought treatment when they had no other choice, often, there was extremely erratic behaviour, which usually meant they were experiencing severe symptoms, such as fainting, panic attacks, self-injury, or ideas of suicide.

*Most people from rural settings seek mental health care after a significant time gap. By the time patients visit professionals or hospitals, their mental condition gets worse than before, and patients become violent and uncontrollable like breaking or damaging things, harming themselves or others. Psychiatrist 1*

However, one mental health advocate also mentioned that most people did not know where to go or whom to seek help from, giving rise to dissatisfaction and negatively impacting their care.

*The idea of mental health is unclear, and people don’t know the difference between a psychiatrist and a psychologist. Also, we are not promoting the importance of mental health and mental health professionals. As people don’t know about the services, they don’t know where to seek mental health care. Mental health advocate 4*

While others needed to become adult to gain agency to seek help or the ability to pay for their treatment.

*My family does not like it and cannot accept it (mental health condition). When the psychiatrist tried to talk to my mother, she became angry. As I earn, I pay for my treatment, so they can’t do anything. They think I have problems with my brain but refuse to acknowledge that I have mental health problems. PWLE 3*

*When I realised that I needed treatment when I was just ten years old, after 14 years of experiencing symptoms such as emotional breakdowns, anxiety attacks, shaking limbs, and suicidal thoughts, I began seeking mental health therapy officially in 2017* [when she became an adult]*. PWLE 3*

### (IV) Affordability of services

Service utilisation and access are also influenced by individuals’ ability to have decision-making power and affordability. Individuals who had used mental health services reported that the treatment cost, in the absence of a personal or social insurance scheme, was a critical barrier to accessing care. The hardest hit were women and people from low- and middle-income groups. This was also made worse if someone lived in a rural area, where they faced additional travel costs, as most mental health professionals were centred in urban areas or the capital. One PWLE said:

*Because the services are based in the capital city, the vast majority of the public is unaware of their existence. It was also challenging for me to go from Khulna (224.6 kilometres from Dhaka) to Dhaka (the capital) for counselling therapy. The sessions were expensive, so I had to stop going and manage my condition myself. PWLE 7*

Although there was agreement that access to mental health treatment was expensive, some psychiatrists felt that affordable treatment was available at government hospitals. One psychiatrist mentioned:

*Overall, health care in our country is affordable. Psychiatric services cost only 10 takas (0.12$) in government institutions, but the services are costlier at private mental healthcare facilities. Psychiatrist 2*

However, even if a person did start treatment, there was often both a lack of trust and a gap in expectation of treatment, with a preference for medication over talking therapies. People did not want to spend money to “just talk” about personal problems. One psychiatrist explained:

*Normally, people assume that why should they pay only to talk. What sort of treatment is that? They aren’t even prepared to comprehend the area of psychology. They believe therapy is ineffective since they were not provided with any medicine. Psychiatrist 2*

Additionally, this mismatch in expectation meant that many did not take up their follow-up visits and complete their treatment as one psychologist suggested:

*Most clients have doubts about talking therapies. They doubt if it would benefit them if their issues were solved; payment is high compared to the doctors. They procrastinate and hesitate to pay the fees for verbal discussion only. We tell them that money is not a matter here and that they should share their issue, but most lose interest. Psychologist 2*

Digital media (television, social media, and web-based newspapers) was seen as a positive development and an important source of information to help raise awareness and increase people’s access to services. One psychiatrist said:

*Nowadays, we can learn about mental health conditions through social media, television media, web-based content, and newspapers. Digital media plays a significant role in increasing mass awareness about mental health in a short time. Psychiatrist 6*

The study reflects that out-of-pocket costs with additional expenses regarding access such as distance and lack of trust to pay for services, act as a significant challenge for Bangladeshi people.

## Theme 2: System-level challenges of mental health services

We asked participants to identify what they thought were the major challenges for the mental health system, and they identified four sub-themes: care pathway, health workforce, governance and financing.

### (I) Care pathway

All stakeholders discussed the importance of a care pathway and the role of primary care in providing mental health services. It was acknowledged that there were significant gaps in how mental health services were configured at a community or sub-district level. Some cautioned that although integrating mental health into primary care services would be essential, this would not be simple due to the high primary care workload and competing health programmes. One psychiatrist said:

*Due to intense pressure and workload at primary settings, upazila health complexes* [primary care facilities] *are overburdened with other kinds of patients, mental health treatments are not provided, and doctors and health workers do not emphasise it. Psychiatrist 1*

The lack of clear referral pathways for specialist care also negatively affected care-seeking behaviours. Many service providers were unaware of the appropriate and available services, which was further complicated by existing power dynamics between different health professionals. As one psychiatrist mentioned:

*The referral system between the psychiatrist and psychologist is not robust. Us psychiatrists are considered a spare team that needs more attention. Psychiatrist 2*

In Bangladesh, people often directly visit a psychiatrist because they seek care late, and not all psychiatrists practice combined drug and talking therapy. Therefore, not all psychiatrists will refer to psychologists. As one psychiatrist said:

*A relatively small percentage of psychiatrists practice combined therapy (medicines and treatments) and refer clients to other mental health professionals when needed.-Psychiatrist 3.*

The psychologists also reiterated the findings in the following way:

*Counselling is a collaborative effort, but a few psychiatrists may have viewed our counsellors as dangerous in the past…. Therapies and medications operate in combination, and when clients’ issues get out of control, they should be referred elsewhere to receive quality service. However, some service providers may perceive it as demeaning, thinking that they do not have the proper expertise. Psychologist 1*

### (II) Health workforce

The lack of job opportunities further demotivates students from pursuing mental health in their careers, and this issue plays a bi-directional relationship in the workforce (Agyapong et al., 2015). Therefore, to reduce the challenges regarding the mental health workforce in LMICs, capacity building of non-specialist healthcare providers by task-sharing approach with proper education, supervision, and partnership with local communities can be an effective solution (Hoeft et al., 2018).

Participants spoke about the critical shortage of mental health specialists, both psychiatrists and psychologists, in Bangladesh. The current supply of a trained workforce does not meet the demand for mental healthcare and negatively affects the quality of service, a crucial factor in addressing the significant treatment gap that exists. Plus, for those who were working in mental health services, there was a lack of proper job opportunities. As one person with a lived experience explained:

*The number of the population dealing with mental health conditions is higher than the available number of psychiatrists in this country. Due to workload, the psychiatrist are unable to provide enough time to the client during a consultation. PWLE 5*

Participants also mentioned little emphasis and exposure to mental health in the undergraduate medical curriculum and awareness of psychiatry as a specialist career. A similar observation was found among nurses and other frontline health cadre training. Besides this, psychologists mentioned that early career psychologists were losing interest in providing counselling services due to low levels of interest among people in seeking mental health treatment and the lack of equal benefits at workplaces. There is a stigma associated with a career in mental health, and the lack of job opportunities means that many doctors and other health-care workers do not wish to pursue further training or, even if trained, decide to change their focus.

*Given the stigma and lack of importance given to mental health in the community, the mental health profession does not receive priority… Sometimes they [psychiatrists] get good facilities in one place but not another. Families also discourage doctors from training [in psychiatry]. Psychologist 4*

Furthermore, stakeholders indicated that the lack of a mental health workforce could be mitigated if the doctors were trained in mental health. However, there are very few opportunities for mental health training, and the undergraduate-level mental health curricula are inadequate for providing needed care. So, when they are posted to public sector facilities, they are unable to detect, manage, and refer patients with mental illness. This is more problematic in rural areas with fewer mental health specialists. One psychologist mentioned:

*In rural areas, there is no mental health specialist available, so the clients go to general physicians seeking mental health care. Most doctors lack knowledge about mental health and don’t receive adequate training on mental health at undergraduate level and even after graduation. So, they fail to detect mental health conditions among general people. Studies have even shown that many of those who committed suicide went to a general practitioner one month before the incident, and the doctor could not catch the mental problem… Psychologist 1.*

Participants who had used services experienced how the mental health professional’s power over them affected their decision-making and led to overmedication and malpractice – an association between high levels of malpractice and low levels of mental health training. Further, professionals’ attitudes and communication skills can be poor; treatment adherence is linked with the judgemental attitude of healthcare professionals. One mental health advocate stated:

*Some service providers tend to judge their clients’ actions and character during the sessions, which later demotivates the clients to continue their sessions. Mental health advocate 5.*

Respondents with a lived experience reported negative personal experiences of healthcare providers’ attitudes that had significantly affected them continuing with their treatment and impacted their care as one described:

*Most service providers cannot connect emotionally with their clients, and we are unsure what to expect. Sometimes, they fail to empathise with what we are going through and the difficulties we are experiencing. PWLE 5*

Participants viewed this because of poor mental health training among the health workforce with increasing reports of malpractice.

### (III) Governance

Participants raised ethical breaches and malpractice among mental health professionals as a factor in eroding public trust in mental health services. There is insufficient regulation and monitoring among different health professionals, which has had a knock-on effect on the ability of different types of mental health professionals to collaborate, develop shared referral pathways, and collectively advocate within the public mental health system. One psychiatrist mentioned:

*In both government and non-government services, there is no platform for experts such as psychologists, occupational therapists, psychiatrists, and social workers. As a result, there is no collective effort to advocate for the job possibilities for these experts in the government health service cadre and other related services. Psychiatrist 4*

Participants also reported that mental health policies were inadequate. One psychiatrist described:

*Mental health policy is not yet defined in our country. In 2011, there was a written proposal to include mental healthcare in UHC. However, it was not clearly defined. But the proposal is still there. Also, the Mental Health Act passed through Cabinet procedure in 2020. However, the Bangladesh government is not waiting for the policy reformulation and taking actions needed to combat the burden of mental health conditions. Psychiatrist 3*

However, another psychiatrist stated that as well as a mental health policy, it was important that there was proper implementation to ensure that the policy goals are achieved.

### (IV) Financing

Most participants reported that the overall resources allocated for mental health services in the public system were inadequate and affected the quality and quantity of services provided. One mental health advocate stated:

*Financing is essential for strengthening the mental health system, yet there is a shortage of funds dedicated to mental health in the total health budget. Sometimes, we try to construct wonderful concepts and plans, but such plans fail to execute owing to a lack of funding. Mental health advocate 1.*

They viewed that the absence of public resources allocated to mental health research meant a lack of evidence regarding what service delivery models could work in Bangladesh. As one psychologist explained:

*The larger picture of mental health challenges in Bangladesh is unaddressed due to a lack of mental health research possibilities. To identify the key issues of mental health challenges in Bangladesh, large-scale research should be conducted, which is not possible due to a lack of funding opportunities from the government level. Psychologist 3*

Further, the lack of health service utilisation data in Bangladesh has meant that treatment gaps cannot be seen easily and advocated for. As one psychiatrist explained:

*Some government health facilities do not have good mental health information and documentation systems. When those statistics are evaluated, it is discovered that Bangladesh has no mental health conditions. Due to communication and traffic difficulties, data is often not even transmitted. This created significant problems since the data would have been utilised for scientific analysis and policy development. Psychiatrist 4*

## Theme 3: Recommendations to strengthen the mental health system

Participants shared their views on the various challenges of the mental health system in Bangladesh and made recommendations.

### (I) Reducing stigma and increasing mental health literacy

Stakeholders identified the need to tackle stigma and address societal superstitions, especially in rural communities, and recommended implementing a national public awareness campaign. They suggested that the campaign should be funded using a public–private partnership model, with leadership and collaboration from volunteer and corporate organisations. The campaign should improve mental health literacy and include information on the prevention of mental health problems and explanations of what mental illness is. They felt that the use of social media would be powerful.

### (II) Strengthening care pathway by increasing access and affordability of services

Another key recommendation was the importance of a low-cost mental health service, which would require the government to introduce subsidies for those with low incomes and a health insurance scheme to reduce the impact of seeking care on an individual’s finances. Universal health coverage of mental health services through primary care was seen to be pivotal, together with strengthening the referral system between healthcare professionals and fostering greater inter-disciplinary and inter-sectoral collaboration. They recommended a service model where mental health professionals were working in primary care, which would improve the quality of referrals to specialist psychiatrists and help use the scarce resources of specialists to effect better. Participants recommended the need to improve the mental health system by developing a national referral system and to upskill all health professionals using educational training programmes.

### (III) Implementation of regulatory mechanisms and capacity building of mental health workforce

Complexity in the governance of the mental health sector was identified as another important issue for the health system. Although Bangladesh introduced the Mental Health Act in 2018 and new mental health policies in 2019, participants felt the lack of a national monitoring body was hindering the implementation of a high-quality mental health service.

Regulation of the mental health workforce, especially counsellors and psychologists, through an accredited regulatory body was felt to be essential to safeguard the public against malpractice. Regulation, however, needed to be linked to a clinical supervision and support system. To make improvements to the mental health workforce, there was a need to introduce psychiatric training at the undergraduate medical, nursing and para-health professional level and improve their ability to deliver behaviour therapy and psychosocial support. There was also the need to retain the existing mental health workforce by creating job opportunities to ensure an adequate number of trained practitioners. They also raised the importance of training other professionals, such as teachers and para-counsellors.

## Discussion

To the best of our knowledge, no prior qualitative research investigating the mental health system in Bangladesh involving diverse groups of mental health stakeholders has been conducted. This study generated findings and recommendations from the perspective of the individual and the system; we found shortfalls and challenges at both levels. Access to mental health services was heavily influenced by individual agencies associated with stigma and low mental health literacy. This was combined with many incurring out-of-pocket expenses and the affordability of mental health services. At a system level, challenges were associated with the absence of care and referral pathways, especially in primary care, shortages in the health workforce, low levels of financing, and the absence of regulation of services and professionals.

Stigma at family and community levels was commonly reported. A similar observation in Indonesia was reported by Subu et al. (2021); who noted stigma about mental illnesses, a lack of family support, shame, rejection and discrimination towards people who sought mental healthcare (Subu et al., 2021). Moreover, such stigmatised beliefs influenced people to seek help from traditional or religious healers, which often complicated their mental health status (Kapur, 1979; Nuri et al., 2018). Previous studies have reported that stigma is linked closely to the lack of mental health literacy among individuals in society (Jorm, 2000). Such issues are common in most LMICs and are identified as a significant obstacle to using mental health services (Ganasen et al., 2008; Ogorchukwu et al., 2016; Begum et al., 2019). Due to an inadequate understanding of mental illness and treatment and the services available, the public resorts to researching their condition and seeking explanations in terms of physical terms (Powell and Clarke, 2006).

Moreover, low literacy dictates attitudes towards medication and treatment, negatively affecting treatment adherence and therapeutic alliance. This was supported by our findings and other researchers (Giasuddin et al., 2014), who also found that treatment delays in Bangladesh were associated with visiting informal care providers such as traditional healers rather than mental health professionals (Giasuddin et al., 2014). This emphasises the importance of raising awareness, reducing stigma, and improving mental health literacy at the population level through mass social movements and awareness programmes. This has also been recommended in similar LMICs (Kumar, 2011; Mascayano et al., 2015; Gwaikolo et al., 2017).

In our study, most stakeholders reported that accessing mental health services was influenced by referrals, which was dependent on the understanding of the mental health professional and the individual. People were confused about how, from whom, and where to seek help (Radez et al., 2021). We found healthcare professionals, family members, and peers who had previous experience seeking mental healthcare influenced the care-seeking behaviour of people who were unfamiliar with services (Aydin et al., 2021). Service use is also influenced by an individual’s ability to have decision-making power and affordability (Hajjaj et al., 2010). The study reports that out-of-pocket costs for services due to travel and lack of trust in public services act as a significant challenge for Bangladeshi people. Digital media and technologies could increase access to mental health services by increasing awareness, offering treatment, and reducing the cost burden (Acharibasam and Wynn, 2018; Berry et al., 2019; Koly et al., 2022b).

The system-level challenges in Bangladesh relate to the need for care pathways, an adequately skilled health workforce, financing and governance. Current mental health services are not integrated as part of universal health coverage, so people living in rural areas are often neglected (Hasan et al., 2021) and incur higher out-of-pocket expenses as they access care privately (Hasan et al., 2021). This skewed distribution of services, with more services in urban areas, is common in similar countries like India, Nepal and Pakistan. Patel and Saxena found that low budgets, an inadequate number of mental health professionals per capita, biomedical framing of mental health problems, a massive shortage of community-based mental healthcare, and infrastructural challenges of primary healthcare centres, were factors that contributed to a failure to ensure universal mental health coverage in LMIC countries (Patel and Saxena, 2019).

We found that the lack of trained mental health workers and career opportunities for them has led to reports of rising workload, malpractice, and inappropriate attitudes towards those using services. This is consistent with other studies where people often experienced the mental health professional’s power over them in decision-making, as well as experiencing malpractice and being overmedicated. There is an association between high levels of malpractice and low levels of mental health training (Mothibi et al., 2019). Current training of doctors is limited to undergraduate training (Koly et al., 2022a), and the lack of job opportunities demotivates doctors from pursuing a career in mental health (Agyapong et al., 2015). In addressing the workforce challenges, potential solutions could include capacity building and task sharing of non-specialist healthcare providers supported by education and supervision and partnerships with local communities (Hoeft et al., 2018). Further solutions could include developing job criteria for mental health professionals at healthcare centres (secondary care) and educational settings.

Complexities in the mental health sector governance were identified as another challenge of the health care system in Bangladesh. Several stakeholders mentioned an absence of central, transparent, accountable processes for monitoring mental health activities and the need for regulation and monitoring at the ministerial level to ensure quality mental health services. This would ensure the protection of human rights and confidentiality of those using services (van Ginneken et al., 2014). A multi-country study in six LMICs reported poor governance as a major challenge in the effective integration of mental health care (Kakuma et al., 2011; Petersen et al., 2017). It has been suggested that the WHO Quality Rights project be adopted and practised to maintain ethical practices and monitor mental health services to protect the rights of the people (World Health, 2012). Moreover, strong leadership and government commitment are required to support the neglected mental health care system (Kakuma et al., 2011).

## Limitations of the study

This study was conducted during the first year of COVID-19 pandemic outbreak; thus, there was an unavoidable bias in selecting participants; participants were required to have access to online platforms. Therefore, the study did not necessarily capture all stakeholders’ voices, especially the lived experience of potentially more vulnerable and marginalised groups. Another limitation was that although the interviews and focus groups were transcribed and translated from Bengali to English, words, and concepts in this process may have changed and lost their actual meaning. However, we mitigated this with the research team, which consisted of bilingual speakers with experience in mental health research and research team discussions. We acknowledge that there may still be cultural issues that would require further assessment, which is not unusual in mental health research. Another significant issue was that we were unable to combine mental service providers such as psychiatrists and psychologists within a group discussion as they had different work patterns during COVID-19 outbreak. However, the individual interviews of psychiatrists helped to get a broader understanding of the mental health sector and the FGDs helped to gain different perspectives within a group setting.

## Recommendations for policymakers

Recommendations included addressing stigma and mental health literacy by raising public awareness of mental health concerns and promoting available services through government-level health education campaigns via respected personalities, which would be feasible strategies to increase mental health literacy. Capacity building across different levels of health workers and discussions on task sharing could be integrated into the policy to reduce malpractice and national oversight and regulation of professional groups and ensure the quality of mental health care. National referral pathways for mental health would progress to equal access across rural and remote areas. In a resource-constrained country setting like Bangladesh, care pathways can be improved by adopting evidence-based mental health programmes, building an alternative mental health workforce, and increasing job opportunities for different-level mental health professionals.

## Conclusion

We found system-level concerns of mental health literacy and social stigma that impacted people’s mental health-seeking behaviour. Stakeholders emphasised the importance of improved mental health care and referral pathways to progress equal access across rural and remote areas. This is an area that is currently understudied in Bangladesh and other LMICs. Other system-level concerns were mental health funding, the lack of professional regulatory bodies and workforce scarcity. Raising public awareness of mental health concerns and promoting available services through government-level health education campaigns via respected personalities would be feasible strategies to increase mental health literacy. Recommendations such as task sharing and capacity building of the different levels of health workers could be integrated into the policy to reduce malpractice and ensure the quality of mental health care.

## Supporting information

Koly et al. supplementary material Koly et al. supplementary material

## Data Availability

The qualitative data used and/or analysed during the current study are available from the corresponding author upon reasonable request.

## Abbreviations

COVID-19: Coronavirus Disease-2019
FGDs: Focus Group Discussions
IDI: In-Depth Interviews
LMICs: Low- and Middle-Income Countries
NGOs: Non-Government Organisations
NIMH: National Institute of Mental Health
PWLE: People With Lived Experiences
UHC: Universal Health Coverage
WHO: World Health Organization

## Supplementary material

The supplementary material for this article can be found at http://doi.org/10.1017/gmh.2024.30.
