# Supplementary material for: Stakeholder perspectives of mental healthcare services in Bangladesh, its challenges and opportunities: a qualitative study
Source: Glob Ment Health (Camb). 2024 Mar 12;11:e37. doi: 10.1017/gmh.2024.30 (PMC10988148; doi:10.1017/gmh.2024.30)
Supplement: Koly et al. supplementary material [file S205442512400030Xsup001.docx]

**SUPPLEMENTARY FILES**

***Supplementary 1:* Interview guidelines**

**Interview Guideline for the people with lived experience (English)**

1. Who did you first contact to seek help with your mental health illness?
2. Why/what prompted you to/ did you contact a mental health service provider?

- Condition
- Psychologist or psychiatrist or others?
- How did you know about them

1. After how many days of feeling unwell or having the problem did you seek help from a mental health service provider? (Time difference to assess the delay in service seeking)
2. For how many days have you been receiving the service or received the service?
3. Have you completed treatment as advised to you by the service? If it is not finished yet, are you regularly taking the service? (to check the adherence)
4. How was your experience with the service provider? (Satisfactory, unpleasant, any other opinion, explore stigma – attitudes here)
5. What were the challenges that you have encountered while seeking mental health care in Bangladesh?

- Family level
- Society level
- Service level
- Cost

1. What are the possible solutions in order to overcome these challenges?

- Family level
- Society level
- Service level
- Cost

1. Do you believe that mental health services should be made widely available? If you don’t believe this why not? If you do think so, why do you think that it should be?
2. How was your mental health condition affected during Covid-19 pandemic?

1. What do you think about the role that technology can play in help improve access and treatment?

**Interview Guideline for the people with lived experience (Bengali)**

১। আপনার মানসিক স্বাস্থ্য সমস্যার জন্য সর্বপ্রথম আপনি কার সাথে যোগাযোগ করেন?

২। কেন বা কোন কারণটির জন্য আপনি মানসিক স্বাস্থ্যসেবা প্রদানকারীর সাথে যোগাযোগ করেছেন?

- আপনার মানসিক স্বাস্থ্য অবস্থার জন্য
- মনোবিদ বা মনোরোগ বিশেষজ্ঞ বা অন্যান্য কারো পরামর্শে
- তাদের সম্পর্কে আপনি কিভাবে জেনেছিলেন?

৩। অসুস্থ বোধ করা বা সমস্যা হওয়ার কতদিন পর আপনি মানসিক স্বাস্থ্যসেবা প্রদানকারীর সাথে যোগাযোগ করেন?

৪। আপনি কতদিন যাবৎ এই স্বাস্থ্যসেবাটি নিচ্ছেন বা নিয়েছিলেন?

৫। আপনি কি মানসিক স্বাস্থ্যসেবা প্রদানকারীর পরামর্শ অনুযায়ী সম্পূর্ণ সেবাটি নিয়েছেন? যদি এখনও না নিয়ে থাকেন, তবে কি নিয়মিত সেবাটি নিচ্ছেন?

৬। স্বাস্থ্যসেবা প্রদানকারী সম্পর্কে আপনার অভিজ্ঞতাটি কেমন ছিল? (সন্তোষজনক, অপ্রীতিকর, অন্য কোন মতামত, সংকীর্ণ মনোভাব)

৭। বাংলাদেশে মানসিক স্বাস্থ্যসেবা নিতে গিয়ে আপনি কি ধরনের সমস্যার মুখোমুখি হয়েছিলেন?

- পারিবারিক পর্যায়ে
- সামাজিক পর্যায়ে
- সেবা দানের ক্ষেত্রে
- খরচের ক্ষেত্রে

৮। এসব সমস্যা কাটিয়ে উঠতে কি কি সমাধান রয়েছে বলে আপনি মনে করেন?

- পারিবারিক পর্যায়ে
- সামাজিক পর্যায়ে
- সেবাদানের ক্ষেত্রে
- খরচের ক্ষেত্রে

৯। আপনি কি মনে করেন, সবার কাছে মানসিক স্বাস্থ্যসেবা ব্যপকভাবে পৌঁছে দেয়া উচিত? আপনি যদি মনে না করেন তবে কেন মনে করেন না? যদি মনে করেন, তবে কেন দেয়া উচিত বলে মনে করেন?

১০। কোভিড-১৯ মহামারী চলাকালীন সময়ে আপনার মানসিক স্বাস্থ্যের অবস্থা কেমন ছিল?

১১। আপনি কি মনে করেন, মানসিক স্বাস্থ্যসেবা উন্নত করতে ডিজিটাল প্রযুক্তি কোন ভূমিকা রাখতে পারে?

Key Informant Interview Guideline for the Psychiatrists (English)

1. For how long have you been in mental health service provision?
2. What kind of patient/people usually come to you for seeking care?
3. How do patients come to you for seeking care?
4. Do you motivate your clients to complete the treatment course? (Got idea of this question after interviewing patient with lived experience)
5. What are the challenges do you think the clients encounter to seek mental health care or to continue mental health care? (Got idea of this question after interviewing patient with lived experience)
6. Do you believe that UHC should include mental health?

If you don’t believe this why not? If you do think so, why do you think that it should be?

1. What does access to mental health treatment look like in your opinion from the patient’s perspective

- Delays to seek care,
- Delay in Identification,
- Delay in Management and referral

1. What do you think are the current challenges with mental health care in Bangladesh? From your experiences

- Overall scenario
- Gaps in services provided
- Gaps in policy

1. What are the solutions in order to overcome these challenges?

- How to reduce the delays in identification, management and referral (from patient level and from providers level)
- Components that should be added in policy and programs
- what are the levels for change?
- integration with other health services i.e. NCD, M&CHC

1. How is mental health care funded? What can be done to reduce the level of out of pocket expenses that people?
2. What do you believe can be learnt from the response to the Covid-19 pandemic that can be helpful in strengthening the mental health system?

1. What do you think about the role that technology can play in help improve access and treatment?

1. Are there any examples of good practice? (probe: integration within different health services, integrating in the primary care, use of technology, working with private and public sector)

Key Informant Interview Guideline for the Psychiatrists (Bengali)

১। আপনি কতদিন ধরে মানসিক স্বাস্থ্যসেবা কাজে নিয়োজিত আছেন?

২। সচরাচর কোন ধরনের মানুষ আপনার কাছে স্বাস্থ্যসেবা নিতে আসে?

৩।স্বাস্থ্যসেবা নেবার জন্য তারা কিভাবে আপনার সাথে প্রথমবার যোগাযোগ করে?

৪। আপনি কি মনে করেন, মানসিক স্বাস্থ্যকে সার্বজনীন স্বাস্থ্যসেবা (ইউ এইচ সি) এর অন্তর্ভুক্ত করা উচিত? যদি মনে না করেন তবে কেন মনে করেন না? যদি মনে করেন, তবে কেন অন্তর্ভুক্ত করা উচিত বলে মনে করেন?

৫। আপনার মতে, রোগীদের মানসিক স্বাস্থ্যসেবা নেবার অভিজ্ঞতা কেমন?

- দেরী করে চিকিৎসা সেবা নেয়া
- দেরীতে সমস্যা চিহ্নিত হওয়া
- দেরীতে স্বাস্থ্যসেবা পাওয়া (চিকিৎসাসেবা ও রেফারেল)

৬। আপনার মতে, বাংলাদেশের বর্তমান মানসিক স্বাস্থ্যসেবা খাতে কি কি সমস্যা রয়েছে? (আপনার অভিজ্ঞতা থেকে বলুন) সামগ্রিক পরিস্থিতি

৭। প্রচলিত সেবাখাতে সমস্যা (নীতিমালার ক্ষেত্রে সমস্যা)

৮। এসব সমস্যা কাটিয়ে উঠতে কি কি সমাধান রয়েছে বলে আপনি মনে করেন?

- কিভাবে রোগ চিহ্নিত করা ও চিকিৎসা সেবা পেতে দেরী হওয়া কমানো যায়
- নতুন আর কি কি বিষয় নীতিমালা ও প্রোগ্রামে যুক্ত করা উচিত
- পরিবর্তনের লেভেলগুলো কি কি?
- অন্যান্য স্বাস্থ্যসেবার সাথে যুক্ত করার মাধ্যমে, যেমন অসংক্রামক ব্যধী , মা ও শিশু স্বাস্থ্য

৯। মানসিক স্বাস্থ্য সেবাখাতে কিভাবে অর্থায়ন করা হয়? ব্যক্তিগত পর্যায় থেকে মানসিক স্বাস্থ্য খাতে কিভাবে কম খরচে বেশি সেবা দেয়া যায় বলে আপনি মনে করেন?

১০। বিশ্বব্যাপী কোভিড-১৯ পরিস্থিতি থেকে আমরা কি কি শিক্ষা গ্রহণ করতে পারি যা মানসিক স্বাস্থ্য খাত জোরদার করতে সাহায্য করবে বলে আপনি মনে করেন?

১১। আপনি কি মনে করেন, ডিজিটাল প্রযুক্তি, মানসিক স্বাস্থ্যসেবা উন্নত করতে কোন ভূমিকা রাখতে পারে?

১২। মানসিক স্বাস্থ্যসেবার ক্ষেত্রে ভালো কোন কাজের উদাহরণ আপনার জানা আছে কি? (যেমন, বিভিন্ন স্বাস্থ্যসেবার সাথে যুক্ত করা, প্রাথমিক স্বাস্থ্যসেবার সাথে যুক্ত করা, প্রযুক্তি ব্যবহার করা, সরকারি ও বেসরকারি বিভাগে কাজ করা)

**Focus Group Discussion Guideline for the Mental Health Advocates and Entrepreneurs (Civil Society) (English)**

1. What types of mental health care your organization provides?
2. What kind of patient/people usually come to you for seeking care?

- Age/gender
- Condition – i.e common or severe mental health disorder, domestic violence etc…
- Pathway/ How do they come to you for seeking care/ contact your organization?
- For how long have you been in service provision

1. Do you motivate your clients to complete the treatment course? (Got idea of this question after interviewing patient with lived experience). If yes – how do you motivate them?
2. What challenges do you think your clients encounter in seeking mental health care or in continuing their mental health care/rehabilitation? (Got idea of this question after interviewing patient with lived experience)
3. Do you believe that UHC should include mental health? If you don’t believe this why not? If you do think so, why do you think that it should be?
4. What does access to mental health treatment look like in your opinion from the patient’s perspective
   - Delays to seek care,
   - Delay in Identification,
   - Delay in Management and referral
5. What do you think are the current challenges with mental health care in Bangladesh? From your experiences

- Overall scenario
- Human resource
- Legislations
- Gaps in services provided
- Gaps in policy

1. What are the solutions in order to overcome these challenges?

- How to reduce the delays in identification, management and referral
- components that should be added in policy and programs
- what are the levels for change
- integration with other health services i.e. NCD, M&CHC

1. How is mental health care funded? What can be done to reduce the level of out of pocket expenses that people?

1. What do you think about the role that technology can play in help improve access and treatment?

1. Are there any examples of good practice happened during Covid-19 that can be helpful in strengthening the mental health system? (probe: integration within different health services, integrating in the primary care, use of technology, working with private and public sector)

**Focus Group Discussion Guideline for the Mental Health Advocates and Entrepreneurs (Civil Society) (Bengali)**

১। আপনার সংস্থাটি কোন ধরনের মানসিক স্বাস্থ্যসেবা দিয়ে থাকে?

- কি ধরনের মানুষ আপনার কাছে স্বাস্থ্যসেবা নিতে আসে
- স্বাস্থ্যসেবা নেবার জন্য তারা কিভাবে আপনার প্রতিষ্ঠানের সাথে প্রথমবার যোগাযোগ করে?
- আপনি কতদিন ধরে এই কাজে নিয়োজিত আছেন?

২। আপনি কি মনে করেন, মানসিক স্বাস্থ্যকে সার্বজনীন স্বাস্থ্যসেবা (ইউ এইচ সি) এর অন্তর্ভুক্ত করা উচিত?

- যদি মনে না করেন তবে কেন মনে করেন না?
- যদি মনে করেন, তবে কেন অন্তর্ভুক্ত করা উচিত বলে মনে করেন?

৩। আপনার মতে, রোগীদের মানসিক স্বাস্থ্যসেবা নেবার অভিজ্ঞতা কেমন?

- দেরী করে চিকিৎসা সেবা নেয়া
- দেরীতে সমস্যা চিহ্নিত হওয়া
- দেরীতে স্বাস্থ্যসেবা পাওয়া (চিকিৎসাসেবা ও রেফারেল)

৪। আপনার মতে, বাংলাদেশের বর্তমান মানসিক স্বাস্থ্যসেবা খাতে কি কি সমস্যা রয়েছে? (আপনার অভিজ্ঞতা থেকে বলুন)

- সামগ্রিক পরিস্থিতি
- প্রচলিত সেবাখাতে সমস্যা
- নীতিমালার ক্ষেত্রে সমস্যা

৫। এসব সমস্যা কাটিয়ে উঠতে কি কি সমাধান রয়েছে বলে আপনি মনে করেন?

- কিভাবে রোগ চিহ্নিত করা ও চিকিৎসা সেবা পেতে দেরী হওয়া কমানো যায়
- নতুন আর কি কি বিষয় নীতিমালা ও প্রোগ্রামে যুক্ত করা উচিত
- পরিবর্তনের লেভেলগুলো কি কি?
- অন্যান্য স্বাস্থ্যসেবার সাথে যুক্ত করার মাধ্যমে, যেমন অসংক্রামক ব্যধী , মা ও শিশু স্বাস্থ্য

৬।মানসিক স্বাস্থ্য সেবাখাতে কিভাবে অর্থায়ন করা হয়? ব্যক্তিগত পর্যায় থেকে মানসিক স্বাস্থ্য খাতে কিভাবে কম খরচে বেশি সেবা দেয়া যায় বলে আপনি মনে করেন?

৭। বিশ্বব্যাপী কোভিড-১৯ পরিস্থিতি থেকে আমরা কি কি শিক্ষা গ্রহণ করতে পারি যা মানসিক স্বাস্থ্য খাত জোরদার করতে সাহায্য করবে বলে আপনি মনে করেন?

৮। আপনি কি মনে করেন, ডিজিটাল প্রযুক্তি, মানসিক স্বাস্থ্যসেবা উন্নত করতে কোন ভূমিকা রাখতে পারে?

৯। মানসিক স্বাস্থ্যসেবার ক্ষেত্রে ভালো কোন কাজের উদাহরণ আপনার জানা আছে কি? (যেমন, বিভিন্ন স্বাস্থ্যসেবার সাথে যুক্ত করা, প্রাথমিক স্বাস্থ্যসেবার সাথে যুক্ত করা, প্রযুক্তি ব্যবহার করা, সরকারি ও বেসরকারি বিভাগে কাজ করা)

**Focus Group Discussion (FGD) Guideline for the Psychologists (English)**

1. For how long have you been in mental health service provision?
2. What kind of patient/people usually come to you for seeking care?
3. How do patients come to you for seeking care?
4. Do you motivate your clients to complete the treatment course? (Got idea of this question after interviewing patient with lived experience)
5. What are the challenges do you think the clients encounter to seek mental health care or to continue mental health care? (Got idea of this question after interviewing patient with lived experience)
6. Do you believe that UHC should include mental health?

If you don’t believe this why not? If you do think so, why do you think that it should be?

1. What does access to mental health treatment look like in your opinion from the patient’s perspective

- Delays to seek care,
- Delay in Identification,
- Delay in Management and referral

1. What do you think are the current challenges with mental health care in Bangladesh? From your experiences

- Overall scenario
- Gaps in services provided
- Gaps in policy

1. What are the solutions in order to overcome these challenges?

- How to reduce the delays in identification, management and referral (from patient level and from providers level)
- Components that should be added in policy and programs
- what are the levels for change
- integration with other health services i.e. NCD, M&CHC

1. How is mental health care funded? What can be done to reduce the level of out of pocket expenses that people?
2. What do you believe can be learnt from the response to the Covid-19 pandemic that can be helpful in strengthening the mental health system?

1. What do you think about the role that technology can play in help improve access and treatment?

1. Are there any examples of good practice? (probe: integration within different health services, integrating in the primary care, use of technology, working with private and public sector)

**Focus Group Discussion (FGD) Guideline for the Psychologists (Bengali)**

১। আপনি কতদিন ধরে মানসিক স্বাস্থ্যসেবা কাজে নিয়োজিত আছেন?

২। সচরাচর কোন ধরনের মানুষ আপনার কাছে স্বাস্থ্যসেবা নিতে আসে?

৩।স্বাস্থ্যসেবা নেবার জন্য তারা কিভাবে আপনার সাথে প্রথমবার যোগাযোগ করে?

৪। আপনি কি মনে করেন, মানসিক স্বাস্থ্যকে সার্বজনীন স্বাস্থ্যসেবা (ইউ এইচ সি) এর অন্তর্ভুক্ত করা উচিত? যদি মনে না করেন তবে কেন মনে করেন না? যদি মনে করেন, তবে কেন অন্তর্ভুক্ত করা উচিত বলে মনে করেন?

৫। আপনার মতে, রোগীদের মানসিক স্বাস্থ্যসেবা নেবার অভিজ্ঞতা কেমন?

- দেরী করে চিকিৎসা সেবা নেয়া
- দেরীতে সমস্যা চিহ্নিত হওয়া
- দেরীতে স্বাস্থ্যসেবা পাওয়া (চিকিৎসাসেবা ও রেফারেল)

৬। আপনার মতে, বাংলাদেশের বর্তমান মানসিক স্বাস্থ্যসেবা খাতে কি কি সমস্যা রয়েছে? (আপনার অভিজ্ঞতা থেকে বলুন) সামগ্রিক পরিস্থিতি

৭। প্রচলিত সেবাখাতে সমস্যা (নীতিমালার ক্ষেত্রে সমস্যা)

৮। এসব সমস্যা কাটিয়ে উঠতে কি কি সমাধান রয়েছে বলে আপনি মনে করেন?

- কিভাবে রোগ চিহ্নিত করা ও চিকিৎসা সেবা পেতে দেরী হওয়া কমানো যায়
- নতুন আর কি কি বিষয় নীতিমালা ও প্রোগ্রামে যুক্ত করা উচিত
- পরিবর্তনের লেভেলগুলো কি কি?
- অন্যান্য স্বাস্থ্যসেবার সাথে যুক্ত করার মাধ্যমে, যেমন অসংক্রামক ব্যধী , মা ও শিশু স্বাস্থ্য

৯। মানসিক স্বাস্থ্য সেবাখাতে কিভাবে অর্থায়ন করা হয়? ব্যক্তিগত পর্যায় থেকে মানসিক স্বাস্থ্য খাতে কিভাবে কম খরচে বেশি সেবা দেয়া যায় বলে আপনি মনে করেন?

১০। বিশ্বব্যাপী কোভিড-১৯ পরিস্থিতি থেকে আমরা কি কি শিক্ষা গ্রহণ করতে পারি যা মানসিক স্বাস্থ্য খাত জোরদার করতে সাহায্য করবে বলে আপনি মনে করেন?

১১। আপনি কি মনে করেন, ডিজিটাল প্রযুক্তি, মানসিক স্বাস্থ্যসেবা উন্নত করতে কোন ভূমিকা রাখতে পারে?

১২। মানসিক স্বাস্থ্যসেবার ক্ষেত্রে ভালো কোন কাজের উদাহরণ আপনার জানা আছে কি? (যেমন, বিভিন্ন স্বাস্থ্যসেবার সাথে যুক্ত করা, প্রাথমিক স্বাস্থ্যসেবার সাথে যুক্ত করা, প্রযুক্তি ব্যবহার করা, সরকারি ও বেসরকারি বিভাগে কাজ করা)

***Supplementary 2:* COREQ (Consolidated criteria for Reporting Qualitative research) Checklist**

| Topic | Item No. | Guide Questions/Description | Remark | Reported on Page No. |
| --- | --- | --- | --- | --- |
| Domain 1: Research team and reflexivity | | | | |
| Personal characteristics | | | | |
| Interviewer/facilitator | 1 | Which author/s conducted the interview or focus group? | Lead author | Methods – 3 |
| Credentials | 2 | What were the researcher’s credentials? E.g. PhD, MD | Author’s credentials reported in end of the manuscript | Manuscript page 16 |
| Occupation | 3 | What was their occupation at the time of the study? | Lead Author: Associate Scientist | Authors credential- 16 |
| Gender | 4 | Was the researcher male or female? | Female: 6, Male:1 | Title page – 1 |
| Experience and training | 5 | What experience or training did the researcher have? | Lead author and other co-authors are experienced in conducting both qualitative research designs. | Method 3 |
| Relationship with participants | | | | |
| Relationship established | 6 | Was a relationship established prior to study commencement? | No relationship with the participants was established before the commencement of the study. | Method -3 |
| Participant knowledge of the interviewer | 7 | What did the participants know about the researcher? e.g. personal goals, reasons for doing the research | The lead author introduced herself to participants stating she is a medical professional and a global mental health researcher, etc. as well as describing the research team, the purpose of the project and answering any questions participants may have had about the study and those involved in it through the email. | Method -3 |
| Interviewer characteristics | 8 | What characteristics were reported about the inter viewer/facilitator? e.g. Bias, assumptions, reasons and interests  in the research topic | The interviewer was a medical professional with a MSc degree in Global Mental Health, involved in multiple mental health research in Bangladesh and also had read the literature on mental health care pathways prior to beginning the study. | Authors credential- 16 |
| Domain 2: Study design | | | | |
| Theoretical framework | | | | |
| Methodological orientation and Theory | 9 | What methodological orientation was stated to underpin the study? e.g.  grounded theory, discourse analysis, ethnography, phenomenology, content analysis | Inductive thematic analysis | Methods – 3 |
| Participant selection | | | | |
| Sampling | 10 | How were participants selected? e.g. purposive, convenience, consecutive, snowball | Purposive selection | Methods – 2 |
| Method of approach | 11 | How were participants approached? e.g. face-to-face, telephone, mail, email | Telephone and followed up by email | Methods – 3 |
| Sample size | 12 | How many participants were in the study? | Total Number of participants= 31 | Result – 4 |
| Non-participation | 13 | How many people refused to participate or dropped out? Reasons? | None of the participants refused to participate in the study. | Method-3 |
| Setting | | | | |
| Setting of data collection | 14 | Where was the data collected? e.g. home, clinic, workplace | Online | Methods – 2 |
| Presence of nonparticipants | 15 | Was anyone else present besides the participants and researchers? | We asked participants to be in a quite room where they would not be disturbed. However, in some cases we could not prevent that a family member occasionally disturbed the interview. | Methods -3 |
| Description of sample | 16 | What are the important characteristics of the sample? e.g. demographic data, date | Reported in the result section | Results – 4  Table 2, Table 3 |
| Data collection | | | | |
| Interview guide | 17 | Were questions, prompts, guides provided by the authors? Was it pilot tested? | All questions are provided in additional file. | Supplementary file 1 |
| Repeat interviews | 18 | Were repeat interviews carried out? If yes, how many? | There was no repeat interview. | Method 3 |
| Audio/visual recording | 19 | Did the research use audio or visual recording to collect the data? | All the interviews and FGDs were recorded through online meeting platforms | Methods 3 |
| Field notes | 20 | Were field notes made during and/or after the interview or focus group? | Other authors took notes during interview and FGDs | Methods 3 |
| Duration | 21 | What was the duration of the interviews or focus group? | The interviews lasted for  40-50 minutes and FGDs lasted for 110-120 minutes on average | Methods - 3 |
| Data saturation | 22 | Was data saturation discussed? | No | N/A |
| Transcripts returned | 23 | Were transcripts returned to participants for comment and/or correction? | No | N/A |
| Domain 3: analysis and findings | | | | |
| Data analysis | | | | |
| Number of data coders | 24 | How many data coders coded the data? | Three | Methods, Data Analysis-4 |
| Description of the coding tree | 25 | Did authors provide a description of the coding tree? | Yes | Figure: 1 |
| Derivation of themes | 26 | Were themes identified in advance or derived from the data? | Derived from the data | Methods -3 |
| Software | 27 | What software, if applicable, was used to manage the data? | No | Methods – 3 |
| Participant checking | 28 | Did participants provide feedback on the findings? | No | N/A |
| Reporting | | | | |
| Quotations presented | 29 | Were participant quotations presented to illustrate the themes/findings?  Was each quotation identified? e.g. participant number | Yes | Results – 4- 13 |
| Data and findings consistent | 30 | Was there consistency between the data presented and the findings? | Yes | Results – 4- 13 |
| Clarity of major themes | 31 | Were major themes clearly presented in the findings? | Yes | Results – 4-13  Figure 1 |
| Clarity of minor themes | 32 | Is there a description of diverse cases or discussion of minor themes? | Yes | Results – 4-13 |

***Supplementary 3:* Additional participant quotes regarding current mental health services in Bangladesh**

| ***Theme 1: Challenges and experiences of using mental health services from an individual level perspective*** | |
| --- | --- |
| ***Subtheme 1.1: Stigma*** | |
| *“Most families of the clients don’t support seeking mental health care. They discourage the client for care seeking and they oppose as they think people will the client ‘Pagol (Mad)’.” Entrepreneur 5* | |
| *“There is lack of awareness in every section. Me and my sisters experienced same difficulties. I realised my problem even it was late. But my sisters accepted that silently and never thought of seeking support.” PWLE 2* | |
| *“My family does not like this thing. They cannot accept it. Psychiatrist tried to make my mother understand.” PWLE 4,* | |
| *“If you talk about financial factors, then there are some specific cultural stigmas limiting service delivery, which is an issue. Firstly, there is a negative attitude among the family members regarding these issues.” Psychiatrist 3* | |
| *“Some people have high stigma which is a great issue,” Psychologist 4* | |
| *“There are some specific cultural stigmas limiting mental health service delivery, which is an issue. Firstly, there is a negative attitude among the family members regarding these issues.” Psychiatrist 3,* | |
| *“If the family is not educated, they cannot understand the mental health problems and they don’t or cannot co-operate in the treatments.” PWLE 5* | |
| *“Females have extreme pressure on them from the family and society. In every situation they are facing difficulties than males. This is affecting their mental health too.” PWLE 4* | |
| *“In our society women are already suffering from a lot of issues and we add more challenges when we ignore the existence of female mental health issues. There is another perspective of the society which is men needs t hide all their pains and feelings. If he does not, he proves himself to be weak.” PWLE 6* | |
| *“There is a taboo in medical students that if they have records of previous mental health issues this may hamper their career in future.” Entrepreneur 8* | |
| *“My father did not understand depression that’s why I could not receive services on that time. My husband understand that I have some problem, but he does not help me that much regarding this matter” PWLE 7* | |
| *“It’s difficult to get something good from this society. If people understand that someone is taking treatment for mental health, they will make him sicker.” PWLE 7* | |
| *“There are also myths and taboos from the people around suggesting that taking too much of medications for mental health illness can deteriorate the mental health further. Sometimes keeping up with follow ups can be difficult.” Psychiatrist 1* | |
| ***Subtheme 1.2: Mental health literacy*** | |
| *“We are very behind in advertisement of the expert psychologists. As people don’t know about the services, they don’t know where to go for mental health care seeking.” Entrepreneur 4* | |
| *“Some people think, they are sharing their intimate and personal issue with the psychologists. They feel guilty about it.” Entrepreneur 1* | |
| *“Very few clients understand that they are having mental health issues and seek care early. Most of them seek care when it is too late and there is no way out.” Entrepreneur 3* | |
| *“Some patients seek care after so long for instance, an individual started experiencing mental health issues at the age of 15 years and now that person is 35 years old, that means since the past twenty years, that person never sought for help and is now visiting us for the first time, the person did not consider the need for help earlier but now they can no longer manage it as that person is facing familial issues, now that person is visiting us.” Psychiatrist 2* | |
| *“If I talk about both young and elderly people, I suppose there remains an average 4-5 years passed after the issue. Some people seek help within 1-2 years……. In the kids’ cases treatment seeking is early due externalising issues like violent behaviour harming others rather than internalising issues.” Psychologist 1* | |
| *“People lose a significant amount of time then seek mental health. This happens a lot that if clients came earlier to seek health, they might not need medicines.  The clients do not seek mental health care until it becomes too necessary.” Psychologist 2* | |
| *“Frankly speaking, it took a lot of time for me to understand that I needed mental health care. It took a lot of time and because of the insufficient/inconvenient health care system of our country I had to face many harassments.” PWLE 2* | |
| *“Most of the patients show good compliance, they keep compliance with us and visit us. Even the patients visiting from outside Dhaka are somewhat are in good compliance. If the patients can’t follow up after six weeks, they still do follow up after ten weeks. In our practice, the patients are in chronic state, and they keep up with it and they regularly follow up with us.’’ Psychiatrist 2* | |
| *“Patients might discontinue treatment when the compliance is poor from both sides and also because patient does not gain enough satisfaction by talking to me or from the treatment. Patients might not also come due to external factors like patient history of substance abuse or no family support.’’ Psychiatrist 2* | |
| *“No, not everyone complete sessions and do not take enough sessions that they need.” Psychologist 1* | |
| *“As the idea of mental health services have not flourished yet, so those who come don't want to pay. Some people who want to pay don’t want to carry on their treatment for a long time. They want fast solutions to their issues.” Psychologist 3* | |
| ***Subtheme 1.3: Access*** | |
| *“In some cases, patients realised after ten to twelve years that they had mental health issues. In case of depression, patients ignore it and somehow try to manage it but after nine months or one year, they visit us when they can no longer manage it.” Psychiatrist 3* | |
| *“Many people lack clear knowledge about counselling, most of them have a mindset that counsellors will solve the issue. But when they are given the whole idea about counselling, they lose interest as they think their issues might not get solved.” Psychologist 5* | |
| *“In Bangladesh the idea about mental health is not clear, still people don’t know the difference between a psychiatrist and a psychologist. Client do not give importance to the treatment when they start to feel better.” Psychologist 4* | |
| *“I realised my problem at the age of 13 or 14 years, but I visited a doctor when I was 19 or 20 years old and became an adult. 5-6 years gap is actually a huge gap.” PWLE 5* | |
| ***Subtheme 1.4: Affordability of services*** | |
| *“When I personally realised there was something wrong with me and wanted to seek mental health care, I contacted a Facebook page and consulted with a psychosocial supporter. Because I couldn't physically see a professional due to financial constraints, my only options were online and telemedicine-based services.” PWLE 7* | |
| *“The cost for the services should be reduced, many students want to take mental health services, but they can not avail because of financial crisis. Many housewives wish but can not avail the services. Because of this hesitation, the mental health status deteriorates.” PWLE 7* | |
| *“Every kind of mental health service cost is out of hand of general people financially. The place where I learn meditation, it costs 3000 taka per hour. Can you imagine? If I ask my sister to learn meditation which will help mentally and refrain her from beating her own child, she will never choose medication for the cost.” PWLE 2* | |
| *“I did not complete all sessions and there are some reasons behind this. Mental health care is very time consuming. These sessions are expensive and for this reason everyone cannot take all the sessions.” PWLE 1* | |
| *“Even I couldn’t realise that the source of problem lies in late past. I just saying I feel restless and depressed. That’s why I wasn’t getting any benefit from mental health care. The psychiatrists in Bangladesh charges very high that I wasn’t prepare financially for taking sessions as I did not see any solution for my problem.” PWLE 3* | |
| *“After starting the session, I realised that it was very costly. That’s why instead of taking session I am trying to control my problems by myself.” PWLE 7* | |
| *“More time and energy are needed for mental health services than physical health. So, I think it is quite normal to suffer more. But the income of the people of Bangladesh is not that much. So, if they are unable to bear the expenses, how you will give services?” PWLE 3* | |
| *^“^Cost can be a reason as the medications have to be used for longer durations, even months or years in case of some patients. So, this can be burden for the patients from the lower socio-economic group. Lack of availability of psychologist prescribed medications in nearby upazillas or under government labels makes it harder for patients to avail the medicines at low costs.” Psychiatrist 1* | |
| *“Most of the clients cannot bear the cost of the sessions. They tend to focus more on the things of life. There is issue of available time also, as they don’t get time after work. Distance also plays a huge role as a barrier.” Psychologist 1* | |
| *“After realising the problem, I contacted with several organisations in online for receiving tele-medicine session as it was impossible for me to take a session in person. But I didn’t receive sufficient response and after 4-5 years, I started to take service formally.” PWLE 7* | |
| *“Nowadays, many services are provided by the experts in social media but it’s totally personally and no one advertise that service unless you know that from the person who received the service.” PWLE 1* | |
| *“Being mentally unstable or having a nervous breakdown impacts physical health very detrimentally. If we are mentally unwell, we will not be physically well too, both are connected. Therefore, there is no chance of giving less importance to mental health.” PWLE 4* | |
| *“When a health insurance structure will be made nationally, mental health services can be included. This will immediately expand the provision of mental health services with subsidiary.”-PWLE 1* | |
| *“If there was an insurance policy for mental health service and care seeking, people might have been seeking care easily.” - Entrepreneur 8.* | |
| ***Theme 2: Challenges of mental health services at system level*** | |
| ***Subtheme 2.1: Care pathway*** | |
| *“In tertiary care facilities, everyday more than 200 patients seek mental health support. And the outdoor patient attending schedule is very limited. We can not provide sufficient time due to huge work load.” – Psychiatrist 3* | |
| *“Due to lack of psychiatrists or mental health professionals at rural areas, it can be difficult for patients to visit Dhaka from other cities like Dinajpur (355.1 Kilometers) or Panchghar (400. 7 Kilometers) for regular follow ups.” Psychiatrist 1* | |
| *“Instead of only centralising this to Dhaka, it should be created based on the districts and upazillas so that all types of people can avail this service.” PWLE 1* | |
| *“Lack of services is not only due to financial factors, changes in administrative structure with lesser expenses can brought about to increase the chances for treatment’’ Psychiatrist 5* | |
| *"Even doctors do not get adequate facilities in terms of their mental health. That’s why they never try to explore patients’ mental health and provide commercial services. Therefore, they do not refer patients to mental health professionals but that does not fulfil the main purpose of the patient.” PWLE 5* | |
| *“Only one or two psychiatrists in Bangladesh who I visited in last 23 years, referred me to the psychologists. It’s a common practice in abroad that, patient will take session from psychologists simultaneously with the medication.” PWLE 5* | |
| *“There is a huge communication gap between the service user and service provider. Clients don’t mention their mental health issues, and the doctors do not explore their mental status. Also the these doctors do not know about the proper referral criteria” Entrepreneur 5* | |
| *“The referral system between the psychiatrist and psychologist is not that strong, us psychiatrists are considered as a spare team which needs nourishment, this concept is still not clear in our country, it is not even clear to the patients, it might also not be clear with the service providers.” Psychiatrist 2* | |
| ***Subtheme 2.2: Health workforce*** | |
| *“We need great amount of human resource in this sector, as there is a lack in the numbers of professionals, the existing service providers cannot provide service properly due to workload.” Entrepreneur 5* | |
| *“Another issue can be manpower. Globally, there is a shortage of manpower especially there are not enough psychiatrists as needed in Bangladesh. Then again, in around ten to twenty years, there won’t be that much of an adequate supply of psychiatrists. Whereas annually only 15/20 graduates come out from three universities combined.” Psychiatrist 1* | |
| *“I have also lived in abroad. But there are significant differences between Bangladesh and abroad countries. I strongly believe that the psychologists of Bangladesh are not well educated.” PWLE 5* | |
| *“I have researched a lot to understand my problems and manage them properly. It seemed to me that psychiatrists in this country are not much up-to-date.” PWLE 5* | |
| *“Some mental health service providers have no respect to the work and the service they are providing.” Entrepreneur 5* | |
| *“Among the psychiatrist I visited in last 23 years in Bangladesh, only one or two have referred me to the psychologists. It’s a common practice abroad that patients will take sessions from psychologists besides taking medication.” PWLE 4* | |
| *“I took only 2-3 sessions from my psychiatrist. I stopped going there for bad experience. 8-9 months after this, I visited another psychiatrist and took some session from her/him.” PWLE 6* | |
| *“I will not say that they go to a point of judgement. But I will definitely say that the counsellor can never connect. The situation through which the patient has gone through and in which situation the patient is saying the words, he/she cannot understand a bit.” PWLE 1* | |
| *“I have seen my cousin receiving mental health service from a psychiatrist in Chittagong. The psychiatrist prescribed sleeping medicines always. The dose was so high that my cousin was lost in the road one day as his/her brain was not working properly. Chances of getting these harmful treatments are still available in Bangladesh.” PWLE 3* | |
| *“I was once prescribed a medicine and when I visited another psychiatrist later, he/she said that I was being prescribed overdose of medicine according to my age.” PWLE 3* | |
| *“Number of mental patients are more than the doctors in this country. That’s why doctors are not providing enough time to the patients.” PWLE 2* | |
| ***Subtheme 2.3 Funding*** | |
| *“At national level health budget, already the budget for health is low, over that in that budget for health, the budget for mental health is lower.” Psychiatrist 1* | |
| ***Subtheme 2.4 Governance*** | |
| *“There is no training institute or licensing organization. This causes professionals to practice individually one their own and sometimes due to wrong treatment choice by the professionals the clients lose time and money.” Psychologist 1* | |
| *“Female Most of the time the counselling is not done in the right way. The number of psychologists who are providing good counselling is very low. As there is no licensing system for providing the service, so there is no way to stop the counselling services provided in a wrong way.” Psychologist 6* | |
| *“Professionals are sometimes unable to provide proper guidance to their patients due to the lack of regulatory bodies. They treat clients only through their personal knowledge and this causes patients to lose money and time in vain, ultimately demotivating the patients, which is not right.” Entrepreneur 5* | |
| *“There is BMDC for doctors, but for psychologists and other experts, there is none, also no law and action.” Entrepreneur 8* | |
| **Theme 3 Participant recommendations to strengthen the mental health care pathway** | |
| *“Awareness in family is really important. If a child faces any harassment somehow, family should take him or her to the counsellor as soon as possible.”* PWLE 2 | |
| *"Awareness and education about mental health need to be strengthened among all the people in our country. Mass people need to be informed politely that Psychiatrists are available for them. Only then people will be interested in receiving services and welfare.”* PWLE 2 | |
| *"As long as the culture of Bangladesh is not getting changed, girls will continue to suffer mentally. Even if they are a victim of any abusing, they won’t be able to tell anyone and as soon as the girl grows up, the pressure of marriage will be there from family and society. Without awareness, people will never realise why mental health services are necessary.”* PWLE 3 | |
| *"If the government wishes, they can easily create awareness all over the country. It is not possible to create awareness on a large scale by private organizations.”* PWLE 3 | |
| *"Where young girls are being abused, in name of affection, people of all ages must be made aware of mental health. It is very crucial for girls to be aware of these issues.”* PWLE 2 | |
| *“Mental health must be a part of the universal health coverage (UHC), but it cannot be done in just a day. It may take time for inclusion and also we need to increase public awareness.”* Psychologist 1 | |
| *“So according to Universal health or WHO, the target is to train the primary level doctors with psychiatric or mental health related curriculum, that’s why the emphasising should be at MBBS doctors at under graduation level. Doctors graduating with MBBS should have good understanding of psychiatry. So that in upazilla level, they can recognise or deal with it at first contact. In that case, even management of mental health treatment will be good.’’* Psychiatrist 1 | |
| *“Teachers should be prioritising the children’s mental health beside physical health and parents should be trained. We have to add life skills into their study materials.”* Psychologist 6 | |
| *“Although many companies offer free services, not everyone knows and does not want to come. Many problems can be reduced if public awareness is strengthened among us.”* Psychologist 2 | |
| *“A lot of public awareness is needed. You need to know when to need help. Going to a counsellor instead of a psychiatrist will not work. Instead of Dhaka-centric work, the whole of Bangladesh-centric work should be done in places where help is needed.”* Psychologist 1 | |
| *“Mental health must be a part of the Universal health coverage, but it cannot be done in just a day. It may take time for inclusion, and we need to increase public awareness.”* Psychologist 8 | |
| *“The way by which Government of Bangladesh has prepared arrangements for HIV Aids or TB, mental health should be spread in the same way.”* PWLE 1 | |
| *“Awareness needs to be developed among people of all categories. Otherwise, the development of mental health welfare will be hampered due to ignorance and lack of cooperation.”* PWLE 5 | |
| *“Government should take steps to make mental health as a mainstream. I cannot share my problem because people will think of me as I am crazy. When this service will be available everywhere, society will not see it negatively.”* PWLE 7, | |
| *“They (psychologist studying in public universities) have studied by our money. Their fees should be made fixed by the Government.”* PWLE 2 | |
| *“May be in future, mental health services can be given for free but that time has not come yet. As the clients for mental health services are already very less, if mental health specialist is enforced to give session at 5-10 taka, they will die in crisis.”* PWLE 3 | |
| *“The families who are not financially solvent, they are educational level are not up to the mark usually. On that case, awareness about mental health and capacity for receiving treatments, both are poor for them.”* PWLE 5 | |
| *“Even though the family is aware, affordability is an important point. Most of the middle-class family cannot spend 2000 taka per session, even though they are willing to take treatment.”* PWLE 6 | |
| *“Governing bodies and organisations like alcohol control authority, Ministry of home affairs, Ministry of Social welfares, Neuro Developmental Disability Prediction Trustee etc. are directly or indirectly are responsible for mental illness or disability rehabilitation. Not waiting for the reformation of Bangladesh government policies, most of them have already started working.’’* Psychiatrist 5 | |
| *“We have to consider the cost issue as well as build a friendly space. Need to reform the structure. Before returning home after rehabilitation, they need to be trained to adapt to society. This is called half-way housing. We need coordination with those who work with mental health internationally.”* Psychologist 6 | |
| *"If we can overcome the shortage of manpower, we can go a long way and provide this service to everyone."* PWLE 1 | |
| *“Training and facilities for doctors and psychologists must be enhanced. This will not happen without the initiatives of the government.”* PWLE 5 | |
| *“We need to create opportunities and provide proper remuneration to the mental health professionals who will provide services at the remote areas.”* Entrepreneur 8 | |
| *“Since psychiatrist or psychologists cannot be quickly made, there needs to be some flexibility so that they can perform counselling till some extent MBBS (general physicians) doctors can provide treatment till some point. But they need to be trained adequately.”* Psychiatrist 1 | |
| *All mental health professionals, all mental health professionals, psychiatrists, psychologists are nothing… all mental health professionals have the scope for capacity building and skill developments which we are not working on.’’* Psychiatrist 4 | |
| *“It is possible to resolve some common disorders at primary level. For this nursing, medical including curriculum of other service providers need to revise in order to put more significance on Psychiatry. Our curriculum is more leaning towards physical health.” Psychiatrist 2* | |
| *“Why are we not including a bigger chapter on psychiatry in MBBS curriculum (undergraduate level)? You cannot just train up if you want. Why is it not done systematically? Psychiatry maybe still somewhat included in nursing curriculum but in other health care curriculum it is not included. They definitely do need training on mental health care.” Psychiatrist 3* | |
| *“There should be a nationally authorised regulatory body and licensing authority and that licensing authority should say that this training is affiliated, and they are capable of providing services as mental health professionals.” Psychiatrist 2* | |
| *“We have to build strong ethics. It need regulation and monitoring to keep good quality of services. If services are provided without supervision for a long time, the quality of services may go downward.” Psychologist 1* | |
| *“To maintain ethics, we should have management for licensing so that unjust manner of the services can be stopped.” Psychologist 6* | |
| *“Licensing needs to be arranged so that false counsellors or psychologists are not created. Covid-19 has created an opportunity for everyone to understand the importance of mental health. That is why many are moving now.” Psychologist 1* | |
| *“There is no good management or supervision system for the rehabilitation system in Bangladesh” Psychologist 4* | |
