# Supplementary material for: Stakeholder perspectives of mental healthcare services in Bangladesh, its challenges and opportunities: a qualitative study
Source: Glob Ment Health (Camb). 2024 Mar 12;11:e37. doi: 10.1017/gmh.2024.30 (PMC10988148; doi:10.1017/gmh.2024.30)
Supplement: Peer review history

# Editor report: Recommendation: Stakeholder perspectives of mental healthcare services in Bangladesh, its challenges and opportunities: a qualitative study — R0/PR2

**DOI:** 10.1017/gmh.2024.30.pr2

**Affiliations:** Columbia University, United States

No accompanying comment.

# Editor report: Decision: Stakeholder perspectives of mental healthcare services in Bangladesh, its challenges and opportunities: a qualitative study — R0/PR3

**DOI:** 10.1017/gmh.2024.30.pr3

**Affiliations:** Johns Hopkins University Bloomberg School of Public Health, United States

No accompanying comment.

# Editor report: Recommendation: Stakeholder perspectives of mental healthcare services in Bangladesh, its challenges and opportunities: a qualitative study — R1/PR5

**DOI:** 10.1017/gmh.2024.30.pr5

**Affiliations:** Columbia University, United States

No accompanying comment.

# Editor report: Decision: Stakeholder perspectives of mental healthcare services in Bangladesh, its challenges and opportunities: a qualitative study — R1/PR6

**DOI:** 10.1017/gmh.2024.30.pr6

**Affiliations:** Johns Hopkins University Bloomberg School of Public Health, United States

No accompanying comment.
